# Hydrogen bis­[2-(4-ammonio­phen­oxy)acetate] triiodide

**DOI:** 10.1107/S1600536810024852

**Published:** 2010-06-30

**Authors:** Wen-Xiang Wang

**Affiliations:** a Ordered Matter Science Research Center, College of Chemistry and Chemical Engineering, Southeast University, Nanjing 211189, People’s Republic of China

## Abstract

In the title compound, C_16_H_19_N_2_O_6_^+^·I_3_^−^, the carboxyl­ate groups of a pair of (4-amino­phen­oxy) acetate ligands are bridged by an H atom in a rather classical configuration. The H atom is located on an inversion center and the pair of carboxyl­ate groups are centrosymmetrically related with an O⋯O distance of 2.494 (5) Å. The I_3_^−^ anion is also located on an inversion center. In the crystal, N—H⋯O and N—H⋯I hydrogen-bond inter­actions build up a three-dimensionnal network.

## Related literature

For dielectric–ferroelectric materials, see: Hang *et al.* (2009 ▶); Li *et al.* (2008 ▶). For related structures, see: Antolic *et al.* (1999 ▶); Bacon *et al.* (1977 ▶); Chen & Mak (1994 ▶); Godzisz *et al.* (2003 ▶); Kay (1977 ▶); Li *et al.* (1998 ▶); McAdam *et al.* (1971 ▶); Pogorzelec & Garbarczyk (2002 ▶); Sridhar *et al.* (2001 ▶); Videnova-Adrabinska *et al.* (2007 ▶); Zhu *et al.* (2002 ▶).
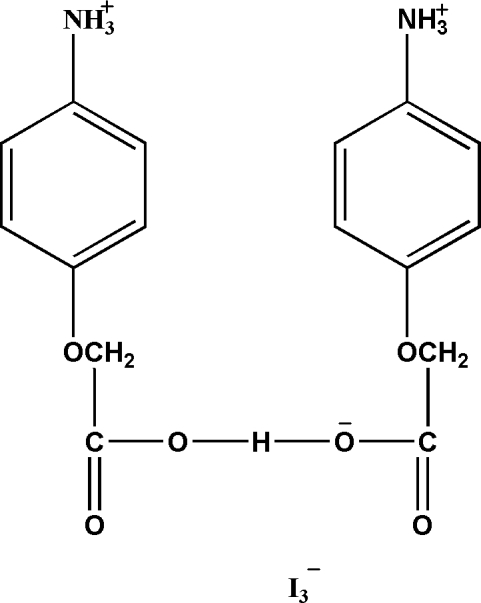


## Experimental

### 

#### Crystal data

C_16_H_19_N_2_O_6_^+^·I_3_^−^
*M* *_r_* = 716.03
Monoclinic, 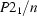

*a* = 5.065 (1) Å
*b* = 13.780 (3) Å
*c* = 14.982 (3) Å
β = 91.45 (3)°
*V* = 1045.4 (4) Å^3^
*Z* = 2
Mo *K*α radiation
μ = 4.52 mm^−1^
*T* = 293 K
0.40 × 0.30 × 0.20 mm

#### Data collection

Rigaku SCXmini diffractometer
Absorption correction: multi-scan (*CrystalClear*; Rigaku, 2005 ▶) *T*_min_ = 0.52, *T*_max_ = 0.58
10680 measured reflections
2408 independent reflections
2182 reflections with *I* > 2σ(*I*)
*R*_int_ = 0.034

#### Refinement

*R*[*F* ^2^ > 2σ(*F* ^2^)] = 0.028
*wR*(*F* ^2^) = 0.062
*S* = 1.14
2408 reflections
124 parameters
H-atom parameters constrained
Δρ_max_ = 0.85 e Å^−3^
Δρ_min_ = −0.75 e Å^−3^

### 

Data collection: *CrystalClear* (Rigaku, 2005 ▶); cell refinement: *CrystalClear*; data reduction: *CrystalClear*; program(s) used to solve structure: *SHELXS97* (Sheldrick, 2008 ▶); program(s) used to refine structure: *SHELXL97* (Sheldrick, 2008 ▶); molecular graphics: *ORTEPIII* (Burnett & Johnson, 1996 ▶), *ORTEP-3 for Windows* (Farrugia, 1997 ▶) and *PLATON* (Spek, 2009 ▶); software used to prepare material for publication: *SHELXL97*.

## Supplementary Material

Crystal structure: contains datablocks I, global. DOI: 10.1107/S1600536810024852/dn2575sup1.cif

Structure factors: contains datablocks I. DOI: 10.1107/S1600536810024852/dn2575Isup2.hkl

Additional supplementary materials:  crystallographic information; 3D view; checkCIF report

## Figures and Tables

**Table 1:** Hydrogen-bond geometry (Å, °)

*D*—H⋯*A*	*D*—H	H⋯*A*	*D*⋯*A*	*D*—H⋯*A*
O3—H3⋯O3^i^	1.25	1.25	2.495 (5)	180
N1—H1*A*⋯I1^ii^	0.89	2.85	3.665 (3)	152
N1—H1*B*⋯O2^iii^	0.89	2.13	2.907 (4)	146
N1—H1*C*⋯O2^iv^	0.89	2.05	2.935 (4)	172

Symmetry codes: (i) 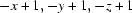; (ii) 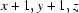; (iii) 
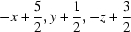
; (iv) 
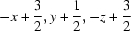
.

**Fig. 1.**
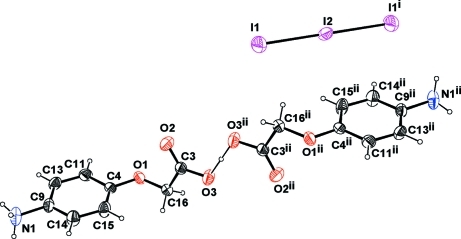
The molecular structure of the title compound, with the atomic numbering scheme. Displacement ellipsoids are drawn at the 30% probability level. H atoms are represented as small spheres of arbitrary radii. [Symmetry codes : (i) -x+1, -y, -z+1; (ii) 1-x, 1-y, 1-z]

**Fig. 2.**
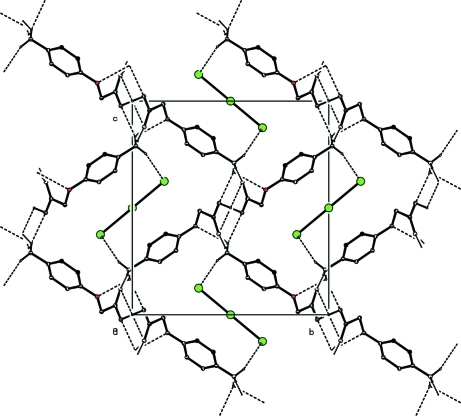
Packing view of the title compound, the a axis. Dashed lines indicate hydrogen bonds. H atoms not involved in hydrogen bondings have been omitted for clarity.

## supplementary crystallographic information

### Comment

We are interested in the dielectric-ferroelectric materials, including organic ligands (Li *et al.*,2008), metal-organic coordination compounds (Hang *et al.*, 2009) and organic-inorganic hybrids. Recent studies have revealed that in amino acid-inorganic acid complexes, when the number of H atoms liberated from the inorganic acid is less than the number of amino acids, the H atom is shared by two amino acids, resulting in short symmetric O—H···O hydrogen bonds, as evidenced in triglycine sulfate (Kay *et al.*, 1977), leading to phase transitions. Thus, we want to find aromatic compounds containing amidogens having dielectric-ferroelectric properties. As part of our ongoing studies, we report here the crystal structure of the title compound.

In the title compound, C_16_H_19_N_2_O_6_^+^.I_3_^-^, the carboxylate groups of a pair of (4-aminophenoxy) acetate are bridged by a proton (Fig. 1) as already observed in many carboxylate derivative (Antolic *et al.*, 1999; Bacon *et al.*,1977; Chen & Mak, 1994; Godzisz *et al.*, 2003; Kay, 1977; Li *et al.*, 1998; McAdam *et al.*,1971; Pogorzelec & Garbarczyk, 2002; Sridhar *et al.*, 2001; Videnova-Adrabinska *et al.*, 2007; Zhu *et al.*, 2002). The proton is located on an inversion center and the pair of carboxylate groups are centrosymmetrically related with an O···O distance of ca 2.494 (5) Å. The two carboxylate frameworks are in the same plane with the largest deviation from the plane being 0.013 (3) Å. This plane is making a dihedral angle of 84.1 (1)° with the phenyl ring.

The anion I_3_^-^ is also located around inversion center. The occurence of N-H···O and N-H···I hydrogen interactions build up a three dimensionnal network (Fig. 2).

### Experimental

ethyl 2-(4-aminophenoxy)acetate (1.95 g) and methanol(30 ml) were added to a round-bottomed flask with a magnetic stirrer bar, then hydrofluoric acid(52%) 2.4 g was added into the mixture. Yellow plate-like crystals of (I) were grown from an ethanol solution of the title compound by slow evaporation at room temperature.

### Refinement

All H atoms attached to C atoms and N atom were fixed geometrically and treated as riding with C—H = 0.93 Å (aromatic) or 0.97 Å (methylene) and N—H = 0.89 Å with U_iso_(H) = 1.2U_eq_(C,N,O).

### Crystal data

**Table:**

C_16_H_19_N_2_O_6_^+^·I_3_^−^	*F*(000) = 672
*M**_r_* = 716.03	*D*_x_ = 2.275 Mg m^−^^3^
Monoclinic, *P*2_1_/*n*	Mo *K*α radiation, λ = 0.71073 Å
Hall symbol: -P 2yn	Cell parameters from 10680 reflections
*a* = 5.065 (1) Å	θ = 3.1–27.5°
*b* = 13.780 (3) Å	µ = 4.52 mm^−^^1^
*c* = 14.982 (3) Å	*T* = 293 K
β = 91.45 (3)°	Prism, yellow
*V* = 1045.4 (4) Å^3^	0.40 × 0.30 × 0.20 mm
*Z* = 2	

### Data collection

**Table:**

Rigaku SCXmini diffractometer	2408 independent reflections
Radiation source: fine-focus sealed tube	2182 reflections with *I* > 2σ(*I*)
graphite	*R*_int_ = 0.034
Detector resolution: 13.6612 pixels mm^-1^	θ_max_ = 27.5°, θ_min_ = 3.1°
ω scans	*h* = −6→6
Absorption correction: multi-scan (*CrystalClear*; Rigaku, 2005)	*k* = −17→17
*T*_min_ = 0.52, *T*_max_ = 0.58	*l* = −19→19
10680 measured reflections	

### Refinement

**Table:**

Refinement on *F*^2^	Primary atom site location: structure-invariant direct methods
Least-squares matrix: full	Secondary atom site location: difference Fourier map
*R*[*F*^2^ > 2σ(*F*^2^)] = 0.028	Hydrogen site location: inferred from neighbouring sites
*wR*(*F*^2^) = 0.062	H-atom parameters constrained
*S* = 1.14	*w* = 1/[σ^2^(*F*_o_^2^) + (0.0183*P*)^2^ + 1.2671*P*] where *P* = (*F*_o_^2^ + 2*F*_c_^2^)/3
2408 reflections	(Δ/σ)_max_ = 0.002
124 parameters	Δρ_max_ = 0.85 e Å^−^^3^
0 restraints	Δρ_min_ = −0.74 e Å^−^^3^

### Special details

**Table:**

Geometry. All esds (except the esd in the dihedral angle between two l.s. planes) are estimated using the full covariance matrix. The cell esds are taken into account individually in the estimation of esds in distances, angles and torsion angles; correlations between esds in cell parameters are only used when they are defined by crystal symmetry. An approximate (isotropic) treatment of cell esds is used for estimating esds involving l.s. planes.
Refinement. Refinement of *F*^2^ against ALL reflections. The weighted *R*-factor *wR* and goodness of fit *S* are based on *F*^2^, conventional *R*-factors *R* are based on *F*, with *F* set to zero for negative *F*^2^. The threshold expression of *F*^2^ > σ(*F*^2^) is used only for calculating *R*-factors(gt) *etc*. and is not relevant to the choice of reflections for refinement. *R*-factors based on *F*^2^ are statistically about twice as large as those based on *F*, and *R*- factors based on ALL data will be even larger.

### Fractional atomic coordinates and isotropic or equivalent isotropic displacement parameters (Å^2^)

**Table:**

	*x*	*y*	*z*	*U*_iso_*/*U*_eq_	
O1	1.2218 (4)	0.67887 (15)	0.58467 (15)	0.0326 (5)	
O2	0.8079 (4)	0.56039 (18)	0.62140 (14)	0.0365 (5)	
O3	0.6743 (4)	0.55858 (17)	0.47780 (15)	0.0367 (5)	
H3	0.5000	0.5000	0.5000	0.044*	
N1	1.1886 (6)	1.0177 (2)	0.7898 (2)	0.0412 (7)	
H1A	1.2238	1.0675	0.7542	0.049*	
H1B	1.3108	1.0144	0.8334	0.049*	
H1C	1.0304	1.0259	0.8132	0.049*	
C3	0.8242 (6)	0.5871 (2)	0.5433 (2)	0.0266 (6)	
C4	1.1951 (6)	0.7628 (2)	0.63381 (19)	0.0253 (6)	
C9	1.1900 (6)	0.9274 (2)	0.73775 (19)	0.0295 (6)	
C11	1.3912 (6)	0.7776 (2)	0.6988 (2)	0.0335 (7)	
H11A	1.5251	0.7321	0.7070	0.040*	
C13	1.3875 (6)	0.8600 (2)	0.7513 (2)	0.0332 (7)	
H13A	1.5172	0.8698	0.7955	0.040*	
C14	0.9956 (6)	0.9123 (3)	0.6741 (2)	0.0369 (7)	
H14A	0.8617	0.9579	0.6662	0.044*	
C15	0.9965 (6)	0.8299 (3)	0.6217 (2)	0.0361 (7)	
H15A	0.8640	0.8199	0.5785	0.043*	
C16	1.0386 (6)	0.6569 (2)	0.5143 (2)	0.0311 (7)	
H16A	1.1324	0.6284	0.4651	0.037*	
H16B	0.9570	0.7166	0.4931	0.037*	
I1	0.53672 (6)	0.162582 (19)	0.624725 (17)	0.05293 (10)	
I2	0.5000	0.0000	0.5000	0.03753 (10)	

### Atomic displacement parameters (Å^2^)

**Table:**

	*U*^11^	*U*^22^	*U*^33^	*U*^12^	*U*^13^	*U*^23^
O1	0.0381 (12)	0.0215 (10)	0.0377 (12)	−0.0003 (9)	−0.0087 (9)	−0.0031 (9)
O2	0.0356 (12)	0.0465 (14)	0.0272 (11)	−0.0053 (10)	−0.0027 (9)	0.0036 (10)
O3	0.0373 (12)	0.0434 (13)	0.0290 (11)	−0.0057 (10)	−0.0059 (9)	−0.0062 (10)
N1	0.0396 (16)	0.0464 (17)	0.0376 (16)	−0.0077 (13)	0.0043 (12)	−0.0193 (13)
C3	0.0285 (15)	0.0233 (14)	0.0280 (15)	0.0060 (11)	−0.0017 (11)	−0.0031 (12)
C4	0.0283 (14)	0.0212 (14)	0.0264 (14)	−0.0055 (11)	0.0002 (11)	0.0026 (11)
C9	0.0344 (16)	0.0309 (16)	0.0234 (14)	−0.0087 (13)	0.0041 (12)	−0.0055 (12)
C11	0.0330 (16)	0.0298 (16)	0.0372 (17)	−0.0007 (13)	−0.0100 (13)	0.0042 (14)
C13	0.0341 (16)	0.0382 (17)	0.0269 (15)	−0.0050 (14)	−0.0084 (12)	0.0009 (13)
C14	0.0332 (17)	0.0375 (18)	0.0398 (18)	0.0090 (14)	−0.0057 (13)	−0.0088 (15)
C15	0.0295 (16)	0.0404 (19)	0.0379 (18)	0.0040 (13)	−0.0117 (13)	−0.0102 (15)
C16	0.0413 (18)	0.0228 (15)	0.0290 (15)	−0.0011 (12)	−0.0050 (13)	−0.0014 (12)
I1	0.0714 (2)	0.04395 (16)	0.04379 (15)	−0.00423 (12)	0.00786 (12)	−0.00539 (11)
I2	0.04291 (18)	0.04059 (18)	0.02891 (16)	0.00398 (13)	−0.00235 (12)	0.00590 (12)

### Geometric parameters (Å, °)

**Table:**

O1—C4	1.380 (4)	C4—C11	1.388 (4)
O1—C16	1.419 (4)	C9—C14	1.369 (4)
O2—C3	1.232 (4)	C9—C13	1.376 (5)
O3—C3	1.287 (4)	C11—C13	1.382 (5)
O3—O3^i^	2.495 (5)	C11—H11A	0.9300
O3—H3	1.2476	C13—H13A	0.9300
N1—C9	1.467 (4)	C14—C15	1.381 (5)
N1—H1A	0.8899	C14—H14A	0.9300
N1—H1B	0.8896	C15—H15A	0.9300
N1—H1C	0.8899	C16—H16A	0.9700
C3—C16	1.522 (4)	C16—H16B	0.9700
C4—C15	1.375 (4)	I1—I2	2.9203 (5)
			
C4—O1—C16	120.3 (2)	C13—C11—C4	120.0 (3)
C3—O3—O3^i^	113.7 (2)	C13—C11—H11A	120.0
C3—O3—H3	113.7	C4—C11—H11A	120.0
C9—N1—H1A	109.4	C9—C13—C11	119.4 (3)
C9—N1—H1B	109.5	C9—C13—H13A	120.3
H1A—N1—H1B	109.5	C11—C13—H13A	120.3
C9—N1—H1C	109.5	C9—C14—C15	120.6 (3)
H1A—N1—H1C	109.5	C9—C14—H14A	119.7
H1B—N1—H1C	109.5	C15—C14—H14A	119.7
O2—C3—O3	125.5 (3)	C4—C15—C14	119.3 (3)
O2—C3—C16	121.7 (3)	C4—C15—H15A	120.3
O3—C3—C16	112.8 (3)	C14—C15—H15A	120.3
C15—C4—O1	125.1 (3)	O1—C16—C3	112.4 (2)
C15—C4—C11	120.2 (3)	O1—C16—H16A	109.1
O1—C4—C11	114.8 (3)	C3—C16—H16A	109.1
C14—C9—C13	120.5 (3)	O1—C16—H16B	109.1
C14—C9—N1	119.1 (3)	C3—C16—H16B	109.1
C13—C9—N1	120.4 (3)	H16A—C16—H16B	107.9

Symmetry codes: (i) −*x*+1, −*y*+1, −*z*+1.

### Hydrogen-bond geometry (Å, °)

**Table:**

*D*—H···*A*	*D*—H	H···*A*	*D*···*A*	*D*—H···*A*
O3—H3···O3^i^	1.25	1.25	2.495 (5)	180
N1—H1A···I1^ii^	0.89	2.85	3.665 (3)	152
N1—H1B···O2^iii^	0.89	2.13	2.907 (4)	146
N1—H1C···O2^iv^	0.89	2.05	2.935 (4)	172

Symmetry codes: (i) −*x*+1, −*y*+1, −*z*+1; (ii) *x*+1, *y*+1, *z*; (iii) −*x*+5/2, *y*+1/2, −*z*+3/2; (iv) −*x*+3/2, *y*+1/2, −*z*+3/2.
